# Identification of Susceptibility Genes for Peritoneal, Ovarian, and Deep Infiltrating Endometriosis Using a Pooled Sample-Based Genome-Wide Association Study

**DOI:** 10.1155/2015/461024

**Published:** 2015-02-04

**Authors:** Bruno Borghese, Jörg Tost, Magalie de Surville, Florence Busato, Frank Letourneur, Françoise Mondon, Daniel Vaiman, Charles Chapron

**Affiliations:** ^1^Institut Cochin, Université Paris Descartes, Sorbonne Paris Cité, CNRS (UMR 8104), 75014 Paris, France; ^2^Inserm, U1016, 75014 Paris, France; ^3^Université Paris Descartes, Sorbonne Paris Cité, Service de Gynécologie Obstétrique 2 et Médecine de la Reproduction, Hôpital Cochin, Hôpitaux Universitaires Paris Centre, AP-HP, 74014 Paris, France; ^4^Laboratoire Epigénétique et Environnement, Centre National de Génotypage, Institut de Génomique/CEA, 91000 Evry, France

## Abstract

Characterizing genetic contributions to endometriosis might help to shorten the time to diagnosis, especially in the most severe forms, but represents a challenge. Previous genome-wide association studies (GWAS) made no distinction between peritoneal endometriosis (SUP), endometrioma (OMA), and deep infiltrating endometriosis (DIE). We therefore conducted a pooled sample-based GWAS and distinguished histologically confirmed endometriosis subtypes. We performed an initial discovery step on 10-individual pools (two pools per condition). After quality control filtering, a Monte-Carlo simulation was used to rank the significant SNPs according to the ratio of allele frequencies and the coefficient of variation. Then, a replication step of individual genotyping was conducted in an independent cohort of 259 cases and 288 controls. Our approach was very stringent but probably missed a lot of information due to the Monte-Carlo simulation, which likely explained why we did not replicate results from “classic” GWAS. Four variants (*rs227849*,* rs4703908*,* rs2479037*, and* rs966674*) were significantly associated with an increased risk of OMA.* Rs4703908*, located close to* ZNF366,* provided a higher risk of OMA (OR = 2.22; 95% CI: 1.26–3.92) and DIE, especially with bowel involvement (OR = 2.09; 95% CI: 1.12–3.91).* ZNF366*, involved in estrogen metabolism and progression of breast cancer, is a new biologically plausible candidate for endometriosis.

## 1. Introduction

Endometriosis is an inflammatory estrogen-driven condition, defined as misplaced endometrium outside of the uterine cavity and causing chronic pelvic pain and infertility [1, 2]. Endometriosis is a major women's health concern that dramatically impairs the quality of life. With a prevalence reaching up to 10% of women of reproductive age, endometriosis has a strong socioeconomic impact [3]. It makes sense to consider endometriosis as a public health priority and to assign substantial human and financial resources to improve the management of patients [4].

Endometriosis is held as a complex heritable trait, with additive genetic effects accounting for about one half of the variance [5]. In recent years, two genome-wide association studies (GWAS) have been conducted in individuals from Japan, Australia, and UK. The first Japanese study reported a significant association of endometriosis with* rs10965235* located in* CDKN2BAS* in 9p21 and with* rs16826658m* in the linkage disequilibrium block including* WNT4* in 1p36 [6].* CDKN2BAS* regulates* P16*, a tumor suppressor genes repressed in endometriosis [7], possibly by promoter hypermethylation [8].* WNT4* has a role in the development of the genitourinary system, steroidogenesis, and folliculogenesis [9, 10]. The second UK/Australian GWAS found an association of endometriosis with* rs12700667* located in 7p15.2 in an intergenic region upstream of* NFE2L3*,* HOXA10*, and* HOXA11* [11]. The role of* HOX* genes in endometriosis-related infertility has been largely debated [7]. After pooling the data from the two studies,* rs12700667* remained significantly associated with endometriosis and also with stages III/IV [12]. Consistent with these associations, the latest GWAS implicated a 150 kb region around* WNT4* that also include* LINC00339* and* CDC42* [13]. An independent set of 305 laparoscopically proven endometriosis patients and 2710 controls confirmed* WNT4*,* CDKN2BAS*, and* FN1* as the first identified common loci for endometriosis [14].

With odds ratios below 1.5, these associations are nonetheless not strong enough to suggest a causal relation or to consider a potential clinical application [15]. This could be due to different genetic backgrounds across populations:* rs10965235* for instance is monomorphic in Caucasians. Most likely, this reflects the heterogeneity of endometriosis [16]. Indeed, endometriosis has inconstant symptoms spanning from no symptoms to severe chronic pelvic pain and infertility [1, 2]. Three distinct clinical forms of endometriosis have been identified. (i) Peritoneal superficial endometriosis (SUP) consists of lesions lying on the surface of the peritoneum or the ovaries. (ii) Endometrioma (OMA) is an ovarian endometriotic “chocolate” cyst. (iii) Deeply infiltrating endometriosis (DIE) comprises lesions infiltrating the* muscularis propria* of structures surrounding the uterus (vagina, bladder, bowel, or ureters) [17]. The phenotypic distinction between SUP, OMA, and DIE may also be raised in terms of their sex hormone responsiveness. SUP, OMA, and DIE have different responsiveness to progesterone [18], differential gene expressions [19, 20], and specific putative variants that may influence one form and not the others [21–23].

Therefore, we initiated a pooled sample-based GWAS with a distinction between SUP, OMA, and DIE. As this was not a classic GWAS approach, we conducted an initial discovery step on 10-individual pools in biological duplicates using the Affymetrix GenChip 250K Nsp chip. After quality control filtering and SNPs ranking based on a false discovery rate (FDR) below 5%, we performed a replication step of individual genotyping to validate the top-ranked SNPs in an independent cohort of 259 endometriosis and 288 controls. Endometriosis was confirmed histologically in cases and invalidated surgically in controls.

## 2. Materials and Methods

### 2.1. Study Population

The data were obtained from 627 unrelated women of Caucasian origin treated in our tertiary referral center for endometriosis and gynecologic conditions. The 319 endometriotic patients underwent a complete surgical resection of all visible lesions allowing confirmation of the diagnosis by expert pathologists in all the cases. We classified the patients into SUP, OMA, and DIE, considering the most severe lesion. DIE was histologically defined when endometriotic lesions involved the* muscularis* of the vagina, the bladder, the intestine, or the ureter [17]. As these three types of lesions were frequently concomitant [24, 25], patients were arbitrarily classified as per the worst findings [26]. Hence, a patient presenting with SUP lesions associated with OMA and DIE was classified as DIE. As well, a patient with OMA and concomitant SUP lesions was classified as OMA, whereas a patient classified as SUP only presented SUP lesions. The 308 controls (CTR) consisted of women without any lesion suggestive of endometriosis, as checked during a comprehensive surgical exploration. Indications for surgery in these patients were infertility work-up, symptomatic uterine fibroids, benign ovarian cysts, or chronic pelvic pain.

The discovery group was composed of 60 cases (20 SUP, 20 OMA, and 20 DIE) and 20 CTR randomly selected from the entire cohort for DNA-pooling experiments. The DIE group included the most severe patients, that is, those presenting at least one lesion infiltrating the bowel and associated bilateral OMAs more than 3 cm. This was done intentionally in order to have a homogeneous group since DIE might encompass a large variety of lesions. The replication cohort was composed of the remaining 259 cases (42 SUP, 121 OMA, and 96 DIE) and 288 CTR. The ethical review board of our center (Comité Consultatif de Protection des Personnes dans la Recherche Biomédicale de Paris-Cochin) approved the study design. All subjects provided written informed consent before entering the study.

### 2.2. DNA Extraction and Quantification, Pool Construction, and SNP Allelotyping

Five millimeters of venous blood were collected from individuals into EDTA tubes. Genomic DNA was subsequently extracted with the MagNa Pure Compact Nucleic Acid Isolation Kit (Roche Applied Science, Indianapolis, IN, USA). DNA was quantified using a spectrophotometer (NanoDrop 1000, Thermo Scientific, Wilmington, DE, USA) and diluted to a concentration of 50 ng/*μ*L. Afterwards each DNA was quantified using fluorimetry (Quant-It DNA Assay Kit, Invitrogen, Carlsbad, CA, USA) and checked for quality using 1% agarose gel electrophoresis. Each pool was composed of 10 samples of 100 ng DNA aliquots from the same category (SUP, OMA, DIE, and CTR). In all, we built eight 10-patient DNA pools, two per category in biological replicates (2 SUP, 2 OMA, 2 DIE, and 2 CTR). DNA quantification and quality were checked several times after the pooling procedure to make sure each pool had the same amount of DNA. Genotyping analysis with GenChip Human Mapping 250K Nsp Array Set (Affymetrix, Santa Clara, CA, USA) was performed for each pool following the manufacturer's guidelines. Briefly, genomic DNAs were restricted with NspI. NspI adaptors were then ligated to restricted fragments and subjected to PCR using the universal primer PCR002 provided by the kit. PCR fragments were then purified and 90 *μ*g used for fragmentation and end-labeling with biotin using Terminal Transferase. Labeled targets were then hybridized overnight to Genechip human 250K NspI array (Affymetrix) at 49°C. Chips were washed on the fluidic station FS450 following specific protocols (Affymetrix) and scanned using the GCS3000 7G. The image was then analyzed with the GCOS software to obtain raw data.

Quality controls were performed using Affymetric GType software and the MPAM algorithm (Modified Partitioning Around Medoids). All samples had a call rate >94% and a detection rate >99%.

### 2.3. Analysis of Microarrays Raw Data

Raw data, presenting as fluorescence intensities for each 25-base perfect match probe (PM) and mismatch probe (MM), were reported and analyzed using Excel 2008 (Microsoft, Redmond, WA). For a given SNP, we computed the amount of fluorescence (*f*) of each allele (*A* and *B*) as *f* = *PM* − *MM*. Allele frequency for allele *A* was estimated by the formula *F*_*A*_ = *f*_*A*_/(*f*_*A*_ + *f*_*B*_) and reciprocally for allele *B*, *F*_*B*_ = *f*_*B*_/(*f*_*A*_ + *f*_*B*_). Then, we calculated the ratio of allele frequencies (*R*) between the cases (SUP, OMA, or DIE) and the controls (CTR). For a given category, since we had 2 pools per category (SUP_1_ and SUP_2_, OMA_1_ and OMA_2_, DIE_1_ and DIE_2_, and CTR_1_ and CTR_2_), we obtained 4 ratios of allele frequencies. As instance for DIE: *R*_1_ = *F*_DIE1_/*F*_CTR1_; *R*_2_ = *F*_DIE1_/*F*_CTR2_; *R*_3_ = *F*_DIE2_/*F*_CTR1_ and *R*_4_ = *F*_DIE2_/*F*_CTR2_. Finally, we calculated the mean of the 4 ratios of allele frequencies and the coefficient of variation (defined as the ratio of the standard deviation to the mean).

### 2.4. Genotypes Calling

SNPs were sorted according to the mean of the four ratios of allele frequencies in each category. We selected the SNPs where the mean of the ratios of allele frequencies was >20. Then, for each selected SNP on a given chromosome, we submitted the mean of the four ratios of allele frequencies and the coefficient of variation to a Monte-Carlo simulation [27]. Briefly, we simulated artificial chromosomes with a number of SNPs identical to that of the chip. For each SNP, we produced random allele frequencies for cases and controls (two of each). These frequencies were used to generate a mean of the four ratios of allele frequencies and a coefficient of variation that were compared to the actual values of each real SNP. This procedure was repeated thousand times, enabling to define a probability of random occurrence of a given pair (mean and coefficient of variation). When this *P* value was <0.05, corresponding to a FDR of 5%, the SNP was considered as significantly biased between cases and controls. For instance, for chromosome 1, four frequencies corresponding to 19,865 SNPs were randomly simulated and used to generate ratios, means, and coefficients of variation, along the lines of what was done with the real dataset. Then, for a real given SNP we performed a test to check if its values (mean and coefficient of variation) could occur and how many times in the artificial database following its random generation. This Monte-Carlo procedure allowed us to eliminate the SNPs whose coefficient of variation was too high and to select the SNPs with a FDR *P* value of 0.05. After this procedure, we obtained a list of 280 SNPs.

### 2.5. Individual Genotyping

To validate high-ranking SNPs from the GWAS, we made another selection by using different criteria. (1) We first selected SNPs from gene regions containing at least 2 SNPs positioned among the top-280 SNPs and/or common to at least two endometriosis subtypes. In this way, we selected 16 SNPs that were individually genotyped by pyrosequencing in 188 cases (42 SUP, 50 OMA, and 96 DIE) and 96 controls from the replication cohort. (2) We also selected the top-5 SNPs, those with a *P* value after Monte-Carlo testing below 0.0001. These SNPs were genotyped in 71 OMA and 192 controls. For technical reasons (degraded DNA), samples used for genotyping the top-5 SNPs were different from those used to test the 16 SNPs first selected. Primers for PCR amplification and pyrosequencing were purchased from BioTeZ (Buch, Germany) (see Supplemental Table 1 in Supplementary Material available online at http://dx.doi.org/10.1155/2015/461024). Regions of interest were amplified using 20 ng of human genomic DNA and 5 pmol of forward and reverse primer, one of them being biotinylated. Reaction conditions were 10x Platinum Taq DNA Polymerase High Fidelity buffer supplemented with 0.5 mM MgCl_2_, 200 mM dNTPs, and 1.5 U Platinum Taq DNA Polymerase High Fidelity (Invitrogen, Cergy Pontoise, France) in a 25 *μ*L volume. The PCR program consisted of a denaturing step of 4 min at 95°C followed by 50 cycles of 30 s at 95°C, 30 s at the respective annealing temperature, and 15 s at 72°C, with a final extension of 4 min at 72°C. Ten *μ*L of each amplification product were purified and rendered single-stranded on a pyrosequencing workstation (Qiagen, Uppsala, Sweden). Beads were released into 12 *μ*L annealing buffer containing 4 pmol of the respective sequencing primer. Primers were annealed to the target by incubation at 80°C for 2 min. Genotyping was carried out on a PyroMark MD system with the PyroGold SQA Reagent Kit (Pyrosequencing) and results were analyzed using the PyroMark MD software (Pyrosequencing). A genetic association study was performed. Statistical analysis was performed using chi-square statistics for assessing single SNP association. A *P* value of <0.05 was considered statistically significant.

## 3. Results

### 3.1. Baseline Characteristics of the Cohorts

Three hundred and nineteen (319) endometriosis cases and 308 disease-free controls were recruited for the study. Basic clinical characteristics of the discovery group (*N* = 60) and the replication cohort (*N* = 567) are described in Supplemental Tables 2 and 3. All patients were of Caucasian origin and presented similar mean age, BMI, parity, gravidity, and infertility. In the discovery group, DIE patients (*N* = 20) were chosen among the patients having a bowel involvement with associated bilateral OMAs, in order to improve the clinical homogeneity of the patients. Indeed, patients with bowel DIE are considered to have the most severe form of endometriosis. In the replicative cohort, DIE group was comprised of patients having lesions of the uterosacral ligaments (*n* = 24), vagina (*n* = 23), bladder (*n* = 12), or intestinal (*n* = 37) lesions. Most of the DIE patients had a bowel involvement (38.5%).

### 3.2. Pooled Sample-Based GWAS

Eight DNA pools of 10 samples each (two per endometriosis subtype and two for controls, in biological duplicate) were hybridized to the Affymetrix 250K chip. For the analysis of pooled sample-based array data, we used Monte-Carlo simulations of the mean of ratios of allelic frequencies and coefficient of variation to rank the 262,000 array SNPs. Two hundred and eighty SNPs were significantly associated with at least one endometriosis group. The complete list of top-ranked SNPs is provided in Supplemental Table 4. The number of significant SNPs was not different according to the subtype (102, 108, and 104 for SUP, OMA, and DIE, resp.). Thirty-two (11.4%) SNPs were common to at least two subtypes (Table 1). Two (0.7%) SNPs (*rs3746192* and* rs776108*) were common to the three subtypes. Fifteen (5.4%) SNPs were common to SUP and OMA, six (2.1%) to SUP and DIE, and nine (3.2%) to OMA and DIE. Among the 280 top-ranked SNPs, SUP had 79 (77.4%) specific SNPs, OMA 82 (75.9%), and DIE 87 (83.7%). This distribution, although not significant (*χ* ^2^ test, *P* = 0.347), suggests that DIE with bowel involvement may be more homogeneous than other forms of the disease.

We examined the expression of the genes corresponding to the 108 top-ranked SNPs in the OMA group according to our transcriptome analysis of OMA [7] (Table 1, Supplemental Table 4). Twenty-seven genes (25.0%) were modified at the expression level (i.e., induced or repressed more than 2-fold), while 16.8% of all the genes were modified in OMA as compared to eutopic endometrium (*P* < 0.001) [7]. This suggests that these 108 variants might affect gene expression in OMA. Such an association is quite original and could give independent clues about the importance of a given gene in a complex disease. Another way to obtain independent clues is the extensive use of bioinformatics that can test whether groups of genes were obtained randomly or not. We performed nonsupervised gene functional clustering separately for each subtypes by using DAVID Bioinformatics Resources (http://david.abcc.ncifcrf.gov/home.jsp). We observed a strong bias of endometriosis-related genes towards transmembrane proteins affecting cell/cell interactions and cell adhesion (Supplemental Table 5).

### 3.3. Individual Genotyping

For this replication step, the average genotyping rate was 97.5%. Hardy-Weinberg equilibrium was confirmed in the control group for all SNPs tested. We performed individual genotyping of 192 cases (96 DIE, 50 OMA, and 46 SUP) and 96 controls for selected SNPs. We first settled on the 16 SNPs that presented the following characteristics: (i) common to the 3 subtypes (*rs3746192, rs776108*) or (ii) common to 2 subtypes and in genes containing other significant SNPs (*rs988147*,* rs227849*,* rs10733833*,* rs322609*,* rs1884779*,* rs4703908*,* rs6946871*,* rs11865033*,* rs247004*,* rs11647011*,* rs7477459*,* rs5913038*,* rs17056959*, and* rs16986515*).* Rs227849* and* rs4703908* were significantly associated with OMA (ORs = 2.31 (1.41–3.78) *P* = 0.003 and 2.22 (1.26–3.92) *P* = 0.009, resp.) (Table 2, Supplemental Table 6).* Rs227849*, introducing A instead of a G at position 44698708 on chromosome 6, is located near* RUNX2*,* SUPT3H*, and* CDC5L*. In the SUP group, a small difference was observed for* rs227849* (*P* = 0.08). In the DIE group,* rs4703908* was significantly associated with a higher risk of bowel endometriosis (OR = 2.09 (1.12–3.91) *P* = 0.012) (Table 2, Supplemental Table 7).* Rs4703908*, related to a G to C change, is located on chromosome 5 in an intronic region near* ZNF366* (zinc finger protein 366). The results for DIE+OMA were not significant for any of the SNPs tested, expect for rs4703907 that was just over the limits of statistical significance (*P* = 0.09). We then focused on OMA, which is regarded as the most well-defined endometriosis phenotype [28]. For this attempt we used even more stringent criteria to select 5 SNPs (*rs11865033*,* rs16913217*,* rs966674*,* rs7333155*, and* rs2479037*). Those criteria were the following: (i) probability of random occurrence in the Monte-Carlo simulation ≤1/1000 and (ii) allelic frequency of the SNP in controls similar to that given by HapMap.* Rs2479037* and* rs966674* were found significantly associated with OMA (OR = 4.36 (1.32–14.43) *P* = 0.005 and 2.95 (1.60–5.42) *P* = 0.002, resp.) (Table 2, Supplemental Table 8).

## 4. Discussion

In this Caucasian population-based study, we performed a pooled sample-based GWAS in endometriosis with a distinction between SUP, OMA, and DIE. Selection of the cases was as rigorous as possible. Systematic histologic confirmation was obtained allowing undoubted classification into one of the three groups. All controls were surgically checked for the absence of endometriosis. We found four new variants (*rs227849*,* rs4703908*,* rs2479037*, and* rs966674*) significantly associated with an increased risk of OMA.* Rs4703908*, located close to* ZNF366,* provided a higher risk of OMA (OR = 2.22 (1.26–3.92)) and DIE, especially with bowel involvement (OR = 2.09 (1.12–3.91)).

This study demonstrated the feasibility of the pooled sample-based method. In “classic” GWAS, the cost associated with the purchase of microarrays and dedicated reagents for tens of thousands of patients put such studies beyond the reach of most research groups. An alternative approach of genotyping pooled instead of individual genomic DNA samples has been developed to reduce the overall cost of GWAS. Our study confirmed the feasibility of DNA pooling on SNP genotyping microarrays and that the pooled sample-based approach presents an attractive and cost-effective alternative to classic GWAS, like other reports [29–32]. The study design has been completed for thousands of dollars whereas individual genotyping requires millions of dollars. For this reasons, other research groups could easily initiate replication and validation of our results. This possibility counterbalances the limitations associated with the pooling strategy, mainly due to the small number of DNA samples. Using 10-individual pools carries a risk of population stratification, as suggested by the discordance in allelic frequencies between our study and the HapMap data for some SNPs. However, two procedures have been used in the present study in order to minimize the biases and remove SNPs for which strong intergroup differences were observed in terms of allelic frequencies: (i) the analysis in parallel of biological duplicates (two pools of 10 patients per condition) and (ii) the Monte-Carlo simulation. In other words, our approach was certainly very stringent but not very sensitive. The SNPs we selected were likely to be valid, but we probably missed some SNPs associated with endometriosis. This point is highlighted by the fact that we did not replicate the SNPs reported by “classic” GWAS [6, 11, 13, 14]. Indeed, it seems difficult to compare the results from “classic” GWAS to our results. First, the SNPs spotted on the Affymetrix chip are not exactly the same as those on the Illumina chip used by other groups. Some genomic regions are not covered by the Affymetrix chip and reciprocally. For example the Affymetrix chip did not cover a genomic region of >5 kb around the variant reported by the Japanese GWAS (rs16826658m). In addition, the patients included in the “classic” GWAS consisted of all-coming endometriosis without distinction between the different phenotypes. Even when a distinction between stages I/II and III/IV was carried out, the rAFS classification was not systematically correlated with the phenotype or the extension of the lesions [33]. In other words, it is quite possible that a patient scored stage I or II might have a bowel endometriosis [34]. Rationale for using the rAFS classification rather than the clinical subtypes (SUP, OMA, and DIE) is truly questionable [35].

It is noteworthy that the four individually validated polymorphisms (*rs4703908, rs227849, rs966674, and rs2479037*) were found in patients suffering from OMA, not from SUP or DIE. In light of the known heterogeneous character of endometriosis, OMA probably represents the most “clear-cut” phenotype of all endometriosis subtypes. Our team recently emphasized this point [28]. Moreover* rs4703908* was significantly associated with bowel endometriosis, a specific form of DIE, considered as the most severe one. This might indicate that bowel DIE could be more genetically driven than the other forms. This observation reinforced the importance of the clinical phenotype of endometriosis, which is, in our opinion, a crucial parameter in GWAS. It might be interesting to collect precise and homogeneous phenotypes, in a multicentric international effort, to conduct targeted GWAS in those populations [17, 34, 36].

Beyond the above-mentioned limitations, we introduced a new tool in the fields of genetics of endometriosis. Two SNPs were common to all subtypes:* rs3746192* and* rs776108*.* Rs3746192* is located in an intronic region near* KCNN1* (potassium intermediate/small conductance calcium-activated channel, subfamily *N*, member 1) on chromosome 19 and related to a G to A change.* KCNN1* is a member of the* KCNN* family of potassium channel genes that are thought to regulate neuronal excitability.* Rs776108*, related to an A to G change, is located in a noncoding region of chromosome 3, near* ROBO2* (roundabout, axon guidance receptor, and homolog 2). ROBO2 is a receptor for SLIT2, known to function in axon guidance and cell migration [37]. Defects in this gene are the cause of vesicoureteral reflux type 2 [38]. With regard to their function,* KCCN1* and* ROBO2* are good candidate genes for endometriosis, since neoneurogenesis frequently occurs in endometriosis [39]. In the OMA group, we have identified four new polymorphisms:* rs4703908*,* rs227849*,* rs966674*, and* rs2479037, *with robust ORs.* Rs2479037* is close to* VTI1A*, possibly involved in colorectal adenocarcinoma [40].* Rs4703908*, associated with both OMA and intestinal DIE, is located near* ZNF366* that plays an important role in regulating the expression of target genes in response to estrogen [41].* ZNF366* could be an independent prognostic factor for breast tumors [42]. ZNF366 could also act as a tumor suppressor in breast cancer development [43, 44]. These findings make it a plausible candidate gene for endometriosis, which is an estrogen-dependent disease [45].

To conclude, the present study clearly indicated some areas of the genome to focus on and confirmed the heterogeneity of endometriosis. The combined use of several polymorphisms identified in this study and in others could eventually permit to define Bayesian probabilities enabling to categorize the patients and predict the risk of endometriosis in future life, as shown on other subjects [46]. Such predictive tool could contribute to a better follow-up of every woman and move towards a person-centered care of infertility and pain associated with endometriosis.

## Supplementary Material

Supplementary Material includes the technical specifications of the primers used for individual genotyping (Supplemental table 1), the baseline characteristics of all participants (Supplemental table 2) and the discovery cohort (Supplemental table 3). Supplemental table 4 provides the top-ranked SNPs significantly associated with at least one endometriosis subtype. Non-supervised gene functional clustering considering genes nearby the 280 top-ranked SNPs is figured in Supplemental table 5. Supplemental tables 6 to 8 provide the results of the replication study: distribution of genotype frequencies in cases and controls for the entire cohort (Supplemental table 6), for the DIE subtypes (Supplemental table 7) and for the OMA subtype (Supplemental table 8).

## Figures and Tables

**Table 1:** Top ranked SNPs significantly associated with at least two endometriosis subtypes. Gene name is provided if the genotyped SNP is located in upstream, 3′UTR, intronic, exonic, 5′UTR, or downstream regions of that gene as annotated by Ensembl Genome Browser GRCh37 (Feb 2009). Inter denotes that a SNP is located in an intergenic region. Chr: chromosome. CV: coefficient of variation. In bold are figured the SNPs common to at least two endometriosis subtypes. Induction ratio is the ratio of gene expression in the endometriotic lesion as compared to the expression in the eutopic endometrium (data from Borghese, 2008). A ratio above 2.0 denotes an upregulation of the gene expression. A ratio below 0.50 denotes a downregulation of the gene expression.

SNP	Chr	SUP			OMA			DIE			Gene (induction ratio)
		Mean	CV	P value	Mean	CV	P value	Mean	CV	P value	
*rs10754006 *	1	111,201	0,064	0,010	195,629	0,302	0,038				RGS1 (2.40) RGS18 (1.0)
*rs776108 *	3	279,764	0,137	0,003	200,537	0,258	0,035	264,901	0,201	0,016	ROBO2 (1.58)
*rs2035669 *	3	223,191	0,339	0,029				153,997	0,256	0,043	GPR156
*rs2200224 *	4				0,008	0,156	0,026	0,008	0,162	0,027	SCFD2 (1.09) RASL11B (2.21)
*rs6850850 *	4				71,599	0,064	0,013	176,564	0,292	0,047	TMEM33 (0.49)
*rs343862 *	4	48,149	0,031	0,013				123,633	0,162	0,031	ARHGAP24
*rs17089182 *	4	0,015	0,068	0,023	0,016	0,054	0,016				Inter
*rs247004 *	5				131,934	0,142	0,017	156,523	0,148	0,012	ACSL6 (1.18)
*rs4703908 *	5				116,523	0,153	0,029	107,713	0,184	0,047	ZNF366 (1.29)
*rs17080695 *	5	73,986	0,124	0,049	45,071	0,050	0,021				MGAT1 (1.08)
*rs17056959 *	6				0,015	0,106	0,044	0,014	0,078	0,024	GPR63 (2.69)
*rs988147 *	6	413,289	0,314	0,005				319,292	0,341	0,020	SUPT3H
*rs227849 *	6	321,093	0,325	0,016	333,485	0,289	0,016				RUNX2 (0.92) SUPT3H (1.20) CDC5L (1.31)
*rs6946871 *	7				172,041	0,165	0,014	173,995	0,354	0,046	SDK1 (0.22)
*rs17138951 *	7	0,004	0,422	0,030	0,005	0,416	0,038				ZNF680 (1.03)
*rs6558435 *	8	236,327	0,468	0,041	181,398	0,150	0,011				ERICH1 (0.93) DLGAP2 (0.89)
*rs10733833 *	10	136,688	0,205	0,033				176,217	0,269	0,027	CTNNA3, LRRTM3
*rs6589535 *	11	132,232	0,277	0,042	78,770	0,133	0,035				Inter
*rs10430848 *	11	254,632	0,439	0,028	148,169	0,187	0,026				TMEM16C (0.69)
*rs2252178 *	12	194,081	0,413	0,035	168,457	0,333	0,044				Inter
*rs1548094 *	14	0,009	0,187	0,023	0,010	0,182	0,024				Inter
*rs11865033 *	16				0,043	0,025	0,009	0,044	0,012	0,000	MAF (1.79)
*rs11647011 *	16	384,064	0,183	0,003	323,569	0,196	0,000				CLEC16A (0.78)
*rs322609 *	16	74,431	0,004	0,000	110,368	0,343	0,042				CDH11 (1.70)
*rs16949415 *	16	0,015	0,099	0,019	0,020	0,098	0,021				WWOX (1.88)
*rs2472694 *	17	82,014	0,159	0,030	81,709	0,223	0,035				GRAP (1.35) PRPSAP2 (0.73) SLC5A10 (1.36)
*rs10871642 *	18				54,143	0,111	0,038	50,092	0,108	0,039	CCDC102B (1.83)
*rs3746192 *	19	93,400	0,173	0,006	78,952	0,149	0,009	97,912	0,063	0,000	KCNN1 (1.02)
*rs6049295 *	20	137,490	0,198	0,007				90,072	0,146	0,015	Inter
*rs1884779 *	20	141,503	0,362	0,031	118,669	0,360	0,035				SULF2 (1.42)
*rs7291901 *	22				0,091	0,030	0,032	0,078	0,029	0,022	XPNPEP3 (0.49)
*rs16986515 *	X	113,443	0,037	0,000				248,589	0,543	0,020	AMELX, ARHGAP6, MSL3L1

**Table 2:** Distribution of genotype and allele frequencies in cases and controls. NA: not applicable, OR: odds ratio, 95% CI: 95% confidence interval, CTR: controls, SUP: superficial peritoneal endometriosis, OMA: endometrioma, and DIG: deep infiltrating endometriosis (DIE) with intestinal involvement. *P* values are for the Pearson's chi-square test. OR and 95% CI are computed for comparison between OMA or DIG and CTR. Phet: *P* value for heterogeneity across discovery and replication steps calculated using the Breslow-Day test. Detailed results for the 21 SNPs tested are available as Supplemental Tables 6 and 8. Detailed results for all subtypes of DIE are available as Supplemental Table 7.

SNP (gene) [chromosome]	Genotypes							Alleles				OR (95% CI)	Phet
	CTR (*N*)			OMA (*N*)			*P* value	CTR (%)		OMA (%)			
Rs227849 (RUNX2, SUPT3H, CDC5L) [6]	AA	AG	GG	AA	AG	GG		A	G	A	G		
	39	39	17	7	27	16	0,003	61.6	38.4	41.0	59.0	2.31 (1.41–3.78)	0,22

Rs4703908 (*ZNF366*) [5]	CC	CG	GG	CC	CG	GG		C	G	C	G		
	64	30	1	24	21	5	0,009	83.2	16.8	69.0	31.0	2.22 (1.26–3.92)	0,28

Rs2479037 (VTI1A) [10]	CC	CT	TT	CC	CT	TT		C	T	C	T		
	5	25	160	0	3	63	0,005	9.2	90.8	2.3	97.7	4.36 (1.32–14.43)	0,34

Rs966674 [5]	CC	CG	GG	CC	CG	GG		C	G	C	G		
	169	22	1	50	17	3	0,002	93.7	6.3	83.6	16.4	2.95 (1.60–5.42)	0,55

	CTR (*N*)			DIG (*N*)			*P*-value	CTR (%)		DIG (%)			

Rs4703908 (*ZNF366*) [5]	CC	CG	GG	CC	CG	GG		C	G	C	G		
	64	30	1	15	22	0	0,012	83.2	16.8	70.3	29.7	2.09 (1.12–3.91)	0,24

## References

[1] Stratton P., Berkley K. J. (2011). Chronic pelvic pain and endometriosis: translational evidence of the relationship and implications. *Human Reproduction Update*.

[2] de Ziegler D., Borghese B., Chapron C. (2010). Endometriosis and infertility: pathophysiology and management. *The Lancet*.

[3] Gao X., Outley J., Botteman M., Spalding J., Simon J. A., Pashos C. L. (2006). Economic burden of endometriosis. *Fertility and Sterility*.

[4] Simoens S., Hummelshoj L., D'Hooghe T. (2007). Endometriosis: cost estimates and methodological perspective. *Human Reproduction Update*.

[5] Treloar S. A., O'Connor D. T., O'Connor V. M., Martin N. G. (1999). Genetic influences on endometriosis in an Australian twin sample. *Fertility and Sterility*.

[6] Uno S., Zembutsu H., Hirasawa A. (2010). A genome-wide association study identifies genetic variants in the *CDKN2BAS* locus associated with endometriosis in Japanese. *Nature Genetics*.

[7] Borghese B., Mondon F., Noël J.-C. (2008). Gene expression profile for ectopic versus eutopic endometrium provides new insights into endometriosis oncogenic potential. *Molecular Endocrinology*.

[8] Borghese B., Barbaux S., Mondon F. (2010). Research resource: genome-wide profiling of methylated promoters in endometriosis reveals a subtelomeric location of hypermethylation. *Molecular Endocrinology*.

[9] Vainio S., Heikkilä M., Kispert A., Chin N., McMahon A. P. (1999). Female development in mammals is regulated by Wnt-4 signalling. *Nature*.

[10] Boyer A., Lapointe É., Zheng X. (2010). WNT4 is required for normal ovarian follicle development and female fertility. *The FASEB Journal*.

[11] Painter J. N., Anderson C. A., Nyholt D. R. (2011). Genome-wide association study identifies a locus at 7p15.2 associated with endometriosis. *Nature Genetics*.

[12] AFS (1985). Revised American Fertility Society classification of endometriosis: 1985. *Fertility and Sterility*.

[13] Albertsen H. M., Chettier R., Farrington P., Ward K. (2013). Genome-wide association study link novel loci to endometriosis. *PLoS ONE*.

[14] Pagliardini L., Gentilini D., Vigano P. (2013). An Italian association study and meta-analysis with previous GWAS confirm WNT4, CDKN2BAS and FN1 as the first identified susceptibility loci for endometriosis. *Journal of Medical Genetics*.

[15] Rahmioglu N., Missmer S. A., Montgomery G. W., Zondervan K. T. (2012). Insights into assessing the genetics of endometriosis. *Current Obstetrics and Gynecology Reports*.

[16] Chapron C., Chopin N., Borghese B. (2006). Deeply infiltrating endometriosis: pathogenetic implications of the anatomical distribution. *Human Reproduction*.

[17] Chapron C., Bourret A., Chopin N. (2010). Surgery for bladder endometriosis: long-term Result s and concomitant management of associated posterior deep lesions. *Human Reproduction*.

[18] Brosens I. A., Brosens J. J. (2000). Redefining endometriosis: is deep endometriosis a progressive disease?. *Human Reproduction*.

[19] Wu Y., Kajdacsy-Balla A., Strawn E. (2006). Transcriptional characterizations of differences between eutopic and ectopic endometrium. *Endocrinology*.

[20] Eyster K. M., Klinkova O., Kennedy V., Hansen K. A. (2007). Whole genome deoxyribonucleic acid microarray analysis of gene expression in ectopic versus eutopic endometrium. *Fertility and Sterility*.

[21] Kitawaki J., Koshiba H., Kitaoka Y. (2004). Interferon-*γ* gene dinucleotide (CA) repeat and interleukin-4 promoter region (−590C/T) polymorphisms in Japanese patients with endometriosis. *Human Reproduction*.

[22] Kang S., Li S. Z., Wang N. (2010). Association between genetic polymorphisms in fibroblast growth factor (FGF)1 and FGF2 and risk of endometriosis and adenomyosis in Chinese women. *Human Reproduction*.

[23] Borghese B., Chiche J.-D., Vernerey D. (2008). Genetic polymorphisms of matrix metalloproteinase 12 and 13 genes are implicated in endometriosis progression. *Human Reproduction*.

[24] Somigliana E., Vercellini P., Gattei U., Chopin N., Chiodo I., Chapron C. (2007). Bladder endometriosis: getting closer and closer to the unifying metastatic hypothesis. *Fertility and Sterility*.

[25] Chapron C., Pietin-Vialle C., Borghese B., Davy C., Foulot H., Chopin N. (2009). Associated ovarian endometrioma is a marker for greater severity of deeply infiltrating endometriosis. *Fertility and Sterility*.

[26] Chapron C., Fauconnier A., Vieira M. (2003). Anatomical distribution of deeply infiltrating endometriosis: surgical implications and proposition for a classification. *Human Reproduction*.

[27] Metropolis N., Ulam S. (1949). The Monte Carlo method. *Journal of the American Statistical Association*.

[28] Borghese B., Santulli P., Héquet D. (2012). Genetic polymorphisms of DNMT3L involved in hypermethylation of chromosomal ends are associated with greater risk of developing ovarian endometriosis. *American Journal of Pathology*.

[29] Meaburn E., Butcher L. M., Schalkwyk L. C., Plomin R. (2006). Genotyping pooled DNA using 100K SNP microarrays: a step towards genomewide association scans. *Nucleic Acids Research*.

[30] Pearson J. V., Huentelman M. J., Halperin R. F. (2007). Identification of the genetic basis for complex disorders by use of pooling-based genomewide single-nucleotide-polymorphism association studies. *The American Journal of Human Genetics*.

[31] Macgregor S., Zhao Z. Z., Henders A., Nicholas M. G., Montgomery G. W., Visscher P. M. (2008). Highly cost-efficient genome-wide association studies using DNA pools and dense SNP arrays. *Nucleic Acids Research*.

[32] Craig J. E., Hewitt A. W., McMellon A. E. (2009). Rapid inexpensive genome-wide association using pooled whole blood. *Genome Research*.

[33] Vercellini P., Fedele L., Aimi G., Pietropaolo G., Consonni D., Crosignani P. G. (2007). Association between endometriosis stage, lesion type, patient characteristics and severity of pelvic pain symptoms: a multivariate analysis of over 1000 patients. *Human Reproduction*.

[34] Dousset B., Leconte M., Borghese B. (2010). Complete surgery for low rectal endometriosis: long-term results of a 100-case prospective study. *Annals of Surgery*.

[35] Laurie C. C., Doheny K. F., Mirel D. B. (2010). Quality control and quality assurance in genotypic data for genome-wide association studies. *Genetic Epidemiology*.

[36] Chapron C., Chiodo I., Leconte M. (2010). Severe ureteral endometriosis: the intrinsic type is not so rare after complete surgical exeresis of deep endometriotic lesions. *Fertility and Sterility*.

[37] Kim M., Roesener A. P., Mendonca P. R. F., Mastick G. S. (2011). Robo1 and Robo2 have distinct roles in pioneer longitudinal axon guidance. *Developmental Biology*.

[38] Lu W., van Eerde A. M., Fan X. (2007). Disruption of ROBO2 is associated with urinary tract anomalies and confers risk of vesicoureteral reflux. *The American Journal of Human Genetics*.

[39] Anaf V., Simon P., El Nakadi I. (2002). Hyperalgesia, nerve infiltration and nerve growth factor expression in deep adenomyotic nodules, peritoneal and ovarian endometriosis. *Human Reproduction*.

[40] Bass A. J., Lawrence M. S., Brace L. E. (2011). Genomic sequencing of colorectal adenocarcinomas identifies a recurrent VTI1A-TCF7L2 fusion. *Nature Genetics*.

[41] Lopez-Garcia J., Periyasamy M., Thomas R. S. (2006). ZNF366 is an estrogen receptor corepressor that acts through CtBP and histone deacetylases. *Nucleic Acids Research*.

[42] Sieuwerts A. M., Ansems M., Look M. P. (2010). Clinical significance of the nuclear receptor co-regulator DC-SCRIPT in breast cancer: an independent retrospective validation study. *Breast Cancer Research*.

[43] Ansems M., Hontelez S., Looman M. W. G. (2010). DC-SCRIPT: nuclear receptor modulation and prognostic significance in primary breast cancer. *Journal of the National Cancer Institute*.

[44] Ansems M., Hontelez S., Karthaus N., Span P. N., Adema G. J. (2010). Crosstalk and DC-SCRIPT: expanding nuclear receptor modulation. *Biochimica et Biophysica Acta—Reviews on Cancer*.

[45] Bulun S. E. (2009). Endometriosis. *The New England Journal of Medicine*.

[46] Ciampolini R., Moazami-Goudarzi K., Vaiman D. (1995). Individual multilocus genotypes using microsatellite polymorphisms to permit the analysis of the genetic variability within and between Italian beef cattle breeds. *Journal of Animal Science*.

