# Supplementary material for: Identification of Susceptibility Genes for Peritoneal, Ovarian, and Deep Infiltrating Endometriosis Using a Pooled Sample-Based Genome-Wide Association Study
Source: Biomed Res Int. 2015 Feb 4;2015:461024. doi: 10.1155/2015/461024 (PMC4334615; doi:10.1155/2015/461024)
Supplement: Supplementary file 1 — Supplementary Material includes the technical specifications of the primers used for individual genotyping (Supplemental table 1), the baseline characteristics of all participants (Supplemental table 2) and the discovery cohort (Supplemental table 3). Supplemental table 4 provides the top-ranked SNPs significantly associated with at least one endometriosis subtype. Non-supervised gene functional clustering considering genes nearby the 280 top-ranked SNPs is figured in Supplemental table 5. Supplemental tables 6 to 8 provide the results of the replication study: distribution of genotype frequencies in cases and controls for the entire cohort (Supplemental table 6), for the DIE subtypes (Supplemental table 7) and for the OMA subtype (Supplemental table 8). [file 461024.f1.pdf]

Supplemental data

Supplemental table 1. Technical specifications of the primers used for individual genotyping

SNP ID	Size	PCR primer forward	PCR primer reverse	Temp PCR (°C)	Pyrosequencing primer(s)
**rs776108** chr3:77,825,927-77,826,729	127	5'-ACCAGGCTAGGCATGCTATA	5'- Biotin-GTCACTTAACAGCAGTGTGTCA	61	ATACCTCTCATTTTGCAG
**rs3746192** chr19:17,946,368-17,946,488	92	5'-ATTCTAGGTGGCATGAGGG	5'- Biotin-CTGGGGAGCAACAGAAGA	56	AGGCTTGTAACCTGGA
**rs988147** chr6:45282108-45282608	169	5' -AGCCATTAAAGAATTTCAAA	5' - Biotine-TTGGATTTTATTCTTGTAATAGG	53	GCAGGGGGTAGAAATG
**rs227849** chr6:44,806,436-44,806,936	94	5' -AGGAAAATAAACATGTGGTTTAAG	5' - Biotine-TCTACCAATATTTTCTTTCGTAGT	56	GGTTTAAGGTCTTTGCAT
**rs10733833** chr10:68,418,227-68,418,727	127	5' -GCCAAAACCAACAGTTCAT	5' - Biotine-GAAAAAAATTGCACCTGTCTC	58	CATGTTTAAAACCTTTCAG
**rs322609** chr16:62,432,604-62,433,104	197	5'-TAGTTGATTTTGCCAACCTG	5'-Biotin-AAATGGGTGACAGAAGTAATAAGA	60	GGTAGCTGTGGGTGGA
**rs1884779** chr20:45,857,969-45,858,469	127	5'-TGGCTATTGGAGTTCTCA	5'-Biotin-CCATCCATCCCAAATAGT	55	AGTGAATTAAGGGCTTGT
**rs4703908** chr5:71,802,353-71,802,853	83	5'- GAAAATGCCCAAGGTGAC	5'-Biotin-GAATGTGGGTGTGTTTTACTCT	52	GTGACAGTGGGCAAA
**rs6946871** chr7:4,037,310-4,037,810	290	5'-ATCAGATAAAATCGGCTTCTGTG	5'-Biotin-TCGGGAAGGTTTTTGTACTTTTG	61	GGAGGAAAGGAAAAGTT
**rs11865033** chr16:78,082,945-78,083,445	123	5'-AATAAACCAAGCCCTGAAAAGTC	5'-Biotin-ACTAAAATCCCCCTTTCCTCCA	61	AAAGTCTCTTCCTATGAGC
**rs247004** chr5:131,372,007-131,372,507	170	5'-GGGGAATTTGTCAGAGATAGGG	5'-Biotin-GGGATCCTCTACCATCCAAATAA	62	GGAAGCCAGACTAGCAG
**rs11647011** chr16:11,115,341-11,115,841	113	5'-TGTGTGTTTATTGGCAATCCC	5'-Biotin-CCCTATGATTTAAGAGGCCAAAGA	61	CCCCTTCCCCTCATC
**rs7477459** chr10:60,671,071-60,671,571	213	5'-GGGTTTCAGGAATTCACTGAGATT	5'-Biotin-TCGCCCTTGTTACATTCTCATTA	63	TCACTGAGATTGGTAGACA
**rs5913038** chrX:78,852,083-78,852,583	107	5'-CATGTTACGGGCTTCAGATTG	5'-Biotin-ATTGAGAATGCACCTGGTCAC	64	TATAAACAATATTAATTTCC
**rs17056959** chr6:97,280,380-97,280,880	181	5'-CRGAACTCCCAGATGTCAAAAAAT	5'-Biotin-TGAGTGTTTGATCCCTGGATTT	61	TTAAATAAAATAATTCATGT
**rs16986515** chrX:11,632,168-11,632,668	125	5'-AGGGGGAAGAAGGTTGGA	5'-Biotin-GAGACCATTGGGCAGGTGTA	56	CTGAGGCCAAAGTCC
**rs11865033** chr16:78,082,945-78,083,445	120	5'-CCAAATCTAATAAACCAAGC	5'- Biotine-CCCCTTTCCTCCATT	51	AAAGTCTCTTCCTATGAGC
**rs16913217** chr11:88,789,821-88,790,321	118	5'- TTTCTTCGAAAGGAGCT	5'- biotine-TCAGAAGGAGATGCATACT	53	TTCGAAAGGAGCTGG
**rs966674** chr5:98,487,305-98,487,805	108	5'-GAGAGGCTAAAATTATTTG	5'- biotine-CTAACCATTTTAAAGGTATC	51	TTTATTTATTGAAAACACAGG
**rs7333155** chr13:26,663,684-26,664,184	209	5'-TAATTTAGAATACTGGTTCAA	5'- Biotine-GAAAGCATGGTGAACATC	51	TCTGAGTCATGTTTTCATC
**rs2479037** chr10:114,586,465-114,586,965	114	5'- TTCTCCTGCACTATTGG	5'- biotine-TGCAGTGGCTTCAAGT	52	CTATTGGCTCACAGTCC

Supplemental table 2. Baseline characteristics of participants. Data are presented as mean ± SD (range) or n (%); NS: not significant; NA: not applicable; [a] Visual analogue scale; [b] Sometimes more than one for the same patient; [c] stage and score according to the revised American Fertility Society (rAFS) classification. P-values are computed with Student-s t-test or Pearson's χ^2-test.

	**Endometriosis N = 319**	**Controls N = 308**	**P-value**
**Age (years)**	31.6 ± 4.9	31.0 ± 5.9	NS
**Height (cm)**	165.3 ± 6.8	165.8 ± 6.1	NS
**Weight (kg)**	59.9 ± 10.2	61.9 ± 11.9	NS
**BMI (kg/m^2)**	21.9 ± 3.6	22.5 ± 4.0	NS
**Parity**	0.2 ± 0.5	0.3 ± 0.8	NS
**Gravidity**	0.5 ± 0.9	0.7 ± 1.3	NS
**Infertility**	125 (39.2)	105 (34.1)	NS
**Preoperative painful symptoms scores[a,b]**			
**Dysmenorrhea**	6.7 ± 2.8	3.8 ± 3.2	<0.001
**Deep dyspareunia**	4.2 ± 3.4	1.5 ± 2.6	<0.001
**Non cyclic chronic pelvic pain**	3.0 ± 3.2	1.7 ± 2.7	<0.001
**Gastro-intestinal symptoms**	3.5 ± 3.6	0.7 ± 2.0	<0.001
**Lower urinary tract symptoms**	1.0 ± 2.3	0.1 ± 0.8	<0.001
**rAFS stage[c]**		NA	
**I**	75 (23.5)		
**II**	70 (21.9)		
**III**	76 (23.8)		
**IV**	98 (30.8)		
**rAFS scores[c]**		NA	
**Adhesions**	13.6 ± 12.3		
**Implants**	17.5 ± 23.1		
**Total**	31.1 ± 30.7		
**Surgical classification**		NA	
**SUP**	62 (19.4)		
**OMA**	141 (44.2)		
**DIE**	116 (36.4)		
**DIE + OMA**	52 (16.3)		
**OMA size (cm)**		NA	
**Right**	3.7 ± 2.3		
**Left**	4.4 ± 3.0		
**OMA laterality**		NA	
**Bilateral**	33/141 (23.3)		
**Right**	46/141 (32.7)		
**Left**	62/141 (44.0)		
**Mean number of DIE lesions**	2.9 ± 2.0	NA	
**Total number of DIE lesions**		NA	
**1**	40/116 (34.2%)		
**2**	26/116 (22.8%)		
**≥ 3**	50/116 (43.0%)		
**Anatomical distribution of DIE**		NA	
**Uterosacral ligament**	82/116 (70.7%)		
**Vagina**	41/116 (35.3%)		
**Bladder**	16/116 (13.8%)		
**Intestine**	57/116 (49.2%)		
**Ureter**	0/116 (0.0%)		
**Worst DIE lesion**		NA	
**Uterosacral ligament**	24/116 (20.7%)		
**Vagina**	23/116 (19.8%)		
**Bladder**	12/116 (10.3%)		
**Intestine**	57/116 (49.2%)		
**Ureter**	0/116 (0.0%)		

Supplemental table 3. Baseline characteristics of the discovery cohort. Data are presented as mean ± SD (range) or n (%); NS: not significant; NA: not applicable; [a] Visual analogue scale; [b] Sometimes more than one for the same patient; [c] stage and score according to the revised American Fertility Society (rAFS) classification. P-values are computed with Student-s t-test or Pearson's χ^2-test.

	SUP (N=20)	OMA (N=20)	DIE (N=20)	P-value
**Age (years)**	32.3 ± 5.9	31.4 ± 4.3	30.9 ± 5.3	NS
**Height (cm)**	164.9 ± 6.4	165.7 ± 5.9	165.1 ± 4.8	NS
**Weight (kg)**	58.9 ± 9.8	59.1 ± 10.9	58.3 ± 7.2	NS
**BMI (kg/m^2)**	22.1 ± 2.6	22.5 ± 3.1	21.7 ± 2.2	NS
**Parity**	0.3 ± 0.5	0.3 ± 0.8	0.3 ± 0.7	NS
**Gravidity**	0.6 ± 1.0	0.7 ± 1.2	0.6 ± 1.2	NS
**Infertility**	7 (35.0)	8 (40.0)	7 (35.0)	NS
**Preoperative painful symptoms scores[a,b]**				<0.01
**Dysmenorrhea**	6.7 ± 2.8	5.2 ± 1.3	7.5 ± 2.9	
**Deep dyspareunia**	4.2 ± 3.4	3.1 ± 1.0	6.1 ± 3.4	
**Non cyclic chronic pelvic pain**	3.0 ± 3.2	2.9 ± 0.3	4.0 ± 2.2	
**Gastro-intestinal symptoms**	3.5 ± 3.6	2.2 ± 1.3	6.9 ± 3.2	
**Lower urinary tract symptoms**	1.0 ± 2.3	0.2 ± 1.2	3.1 ± 2.2	
**rAFS stage[c]**				< 0.001
**I**	9 (45.0)	0 (0.0)	0 (0.0)	
**II**	11 (55.0)	0 (0.0)	0 (0.0)	
**III**	0 (0.0)	14 (70.0)	8 (40.0)	
**IV**	0 (0.0)	6 (30.0)	12 (60.0)	
**OMA size (cm)**	NA			NS
**Right**		4.5 ± 2.2	3.9 ± 2.0	
**Left**		4.6 ± 3.0	4.5 ± 3.9	
**OMA laterality**	NA			< 0.01
**Bilateral**		0/20 (0.0)	20/20 (100.0)	
**Right**		10/20 (50.0)	0/20 (100.0)	
**Left**		10/20 (50.0)	0/20 (100.0)	
**Mean number of DIE lesions**	NA	NA	3.6 ± 1.2	NA
**Anatomical distribution of DIE**	NA	NA		NA
**Uterosacral ligament**			18/20 (90.0%)	
**Vagina**			15/20 (75.0%)	
**Bladder**			0/20 (0.0%)	
**Intestine**			20/20 (100.0%)	
**Ureter**			0/116 (0.0%)	
**Worst DIE lesion**	NA	NA		NA
**Uterosacral ligament**			0/20 (0.0%)	
**Vagina**			0/20 (0.0%)	
**Bladder**			0/20 (0.0%)	
**Intestine**			20/20 (100%)	
**Ureter**			0/20 (0.0%)	

Supplemental table 4. Top ranked SNPs significantly associated with at least one endometriosis subtype. Gene name is provided if the genotyped SNP is located in upstream, 3'UTR, intronic, exonic, 5'UTR, or downstream regions of that gene as annotated by Ensembl Genome Browser GRCh37 (Feb 2009). Inter denotes a SNP is located in an intergenic region. Chr: chromosome. CV: coefficient of variation. In bold are figured the SNPs common to at least two endometriosis subtypes. Induction ratio is the ratio of gene expression in the endometrioma as compared to the expression in the eutopic endometrium (data from Borghese, 2008). A ratio above 2.0 denotes an upregulation of the gene expression. A ratio below 0.50 denotes a downregulation of the gene expression.

SNP	Chr	SUP			OMA			DIE			Gene (induction ratio)
		mean	CV	P-value	mean	CV	P-value	mean	CV	P-value	
**rs10754006**	**1**	**111,201**	**0,064**	**0,010**	**195,629**	**0,302**	**0,038**				**RGS1 (2.40) RGS18 (1.0)**
SNP_A-1916487	1							0,004	0,247	0,019	IL23R
rs501982	1							0,011	0,061	0,014	FAM5C
rs7549603	1							0,032	0,041	0,032	SCP2
rs3124815	1							0,062	0,025	0,047	TPR
rs10925310	1							84,530	0,026	0,001	RYR2
rs6426015	1							85,534	0,100	0,027	ZC3H12A
rs6588264	1	27,114	0,029	0,030							SERBP1
rs11162229	1	70,223	0,110	0,047							ST6GALNAC5
rs749069	1				57,356	0,038	0,007				DAB1 (0.30)
rs1479739	1				156,083	0,217	0,030				inter
rs17503170	2							0,008	0,079	0,010	inter
rs12469375	2							202,299	0,137	0,005	ERBB4
rs17047369	2							202,776	0,168	0,012	inter
rs16838630	2							150,828	0,212	0,043	MDH1B
rs10206624	2							63,091	0,036	0,007	C1D, ETAA1
rs2041753	2	0,004	0,294	0,020							IL1RL2
rs6717212	2	270,955	0,082	0,000							MKI67IP
rs9636439	2	241,048	0,300	0,019							SMEK2
rs1967351	2	138,594	0,110	0,020							CERKL
rs736711	2	147,416	0,057	0,003							inter
rs4143154	2	90,376	0,122	0,035							RSAD2
rs1526383	2				72,101	0,065	0,011				inter
rs2220127	2				97,147	0,094	0,018				FLJ39502 (2.30)
rs6748466	2				120,297	0,140	0,023				ZNF804A (0.72)
**rs776108**	**3**	**279,764**	**0,137**	**0,003**	**200,537**	**0,258**	**0,035**	**264,901**	**0,201**	**0,016**	**ROBO2 (1.58)**
**rs2035669**	**3**	**223,191**	**0,339**	**0,029**				**153,997**	**0,256**	**0,043**	**GPR156**
rs6786265	3							0,014	0,088	0,026	AMOTL2, RYK
rs698208	3							0,027	0,052	0,042	SUMF1
rs4586824	3							0,033	0,024	0,007	FAM19A1
rs870852	3							78,914	0,118	0,036	SCHIP1
rs7427791	3	0,007	0,113	0,010							GBE1
rs1016449	3	0,016	0,015	0,004							FHIT
rs1259334	3	164,693	0,325	0,047							FSTL1
rs17620655	3				0,005	0,238	0,016				ZNF659 (1.51)
rs1527561	3				43,188	0,008	0,000				MYRIP (1.25)
rs10513504	3				75,784	0,080	0,025				PTX3 (3.04) VEPH1 (0.23)
rs17514779	3				89,609	0,026	0,001				CD200 (0.74) BTLA (1.07)
rs2736779	3				179,832	0,308	0,041				FHIT (1.65)
**rs2200224**	**4**				**0,008**	**0,156**	**0,026**	**0,008**	**0,162**	**0,027**	**SCFD2 (1.09) RASL11B (2.21)**
**rs6850850**	**4**				**71,599**	**0,064**	**0,013**	**176,564**	**0,292**	**0,047**	**TMEM33 (0.49)**
**rs343862**	**4**	**48,149**	**0,031**	**0,013**				**123,633**	**0,162**	**0,031**	**ARHGAP24 (4.08)**
**rs17089182**	**4**	**0,015**	**0,068**	**0,023**	**0,016**	**0,054**	**0,016**				**inter**
rs6531411	4							0,006	0,132	0,012	CENTD1
rs1122675	4							0,007	0,146	0,022	inter
rs1480721	4							0,016	0,057	0,019	BMP2K, PAQR3
rs17030252	4							51,374	0,060	0,026	EMCN, PPP3CA
rs17012007	4	0,007	0,158	0,017							PTPN1
rs2635208	4	0,012	0,073	0,017							NDST3
rs17685980	4	114,756	0,132	0,031							NDST4
rs6531296	4	115,604	0,163	0,033							PCDH7
rs36054314	4	97,888	0,108	0,027							PRDM8, ANTXR2
rs16845668	4	99,890	0,083	0,019							DCK
rs4437257	4	76,230	0,091	0,023							MGC46496
rs2622628	4	0,015	0,087	0,040							ABCG2
rs7690923	4	55,918	0,052	0,019							TBC1D19, STIM2

rs17832300	4				67,467	0,080	0,029				C4orf14 (0.92) IGFBP7 (1.32) POLR2B (0.82)
rs11733111	4				77,208	0,107	0,044				CLCN3 (0.47)
rs1480713	4				83,776	0,084	0,025				BMP2K (0.73) PAQR3 (0.35)
rs2537869	4				101,788	0,141	0,031				KIT (5.35)
**rs247004**	**5**				**131,934**	**0,142**	**0,017**	**156,523**	**0,148**	**0,012**	**ACSL6 (1.18)**
**rs4703908**	**5**				**116,523**	**0,153**	**0,029**	**107,713**	**0,184**	**0,047**	**ZNF366 (1.29)**
**rs17080695**	**5**	**73,986**	**0,124**	**0,049**	**45,071**	**0,050**	**0,021**				**MGAT1 (1.08)**
rs10042444	5							0,008	0,162	0,024	SH3RF2
rs323763	5							0,024	0,019	0,004	MGC33214
rs966674	5							0,048	0,008	0,006	inter
rs10940176	5							202,000	0,401	0,048	PIK3R1
rs251529	5							204,727	0,201	0,023	MOCS2, FST
rs7733760	5							110,367	0,167	0,044	inter
rs1423113	5	172,857	0,308	0,039							ZFYVE16
rs10512928	5	58,552	0,041	0,010							ADCY2
rs16899953	5	40,179	0,048	0,038							MGC23909
rs17147703	5	0,007	0,225	0,027							FTMT
rs1345683	5				0,006	0,090	0,001				FLJ43080 (0.24)
rs2619861	5				0,024	0,017	0,001				C5orf23 (1.21)
rs6862278	5				100,754	0,112	0,019				DOCK2 (1.96)
rs10053139	5				102,299	0,093	0,018				CDH12 (0.37)
**rs17056959**	**6**				**0,015**	**0,106**	**0,044**	**0,014**	**0,078**	**0,024**	**GPR63 (2.69)**
**rs988147**	**6**	**413,289**	**0,314**	**0,005**				**319,292**	**0,341**	**0,020**	**SUPT3H**
**rs227849**	**6**	**321,093**	**0,325**	**0,016**	**333,485**	**0,289**	**0,016**				**RUNX2 (0.92) SUPT3H (1.20) CDC5L (1.31)**
rs4959712	6							0,009	0,048	0,006	MYLK4
rs2571587	6							0,010	0,130	0,027	LAMA2
rs9322830	6							0,019	0,014	0,001	LIN28B
rs6940248	6							0,023	0,047	0,028	MAN1A1
rs1615962	6							283,962	0,240	0,014	inter
rs16894463	6							296,866	0,225	0,011	NCR2
rs9296749	6							33,257	0,043	0,033	C6orf142 TINAG
rs12111005	6							33,139	0,048	0,032	PRL, HDGFL1
rs4708515	6	111,556	0,123	0,020							THBS2, SMOC2
rs12524898	6	264,808	0,424	0,030							inter
rs9497102	6	53,066	0,053	0,016							UTRN
rs3789771	6				0,021	0,030	0,005				EFHC1 (0.31) TRAM2 (1.40)
rs6570813	6				90,675	0,112	0,031				SASH1 (0.74)
rs9401519	6				113,431	0,144	0,014				HSF2 (1.03)
rs9321325	6				115,893	0,192	0,049				FBXL4 (0.79)
rs10947850	6				126,780	0,228	0,047				EDN1 (1.86) HIVEP1 (1.03)
**rs6946871**	**7**				**172,041**	**0,165**	**0,014**	**173,995**	**0,354**	**0,046**	**SDK1 (0.22)**
**rs17138951**	**7**	**0,004**	**0,422**	**0,030**	**0,005**	**0,416**	**0,038**				**ZNF680 (1.03)**
rs4478510	7							0,011	0,141	0,030	C7orf53, TMEM168
rs6977949	7							112,668	0,104	0,013	KCND2
rs17148913	7							24,001	0,040	0,038	inter
rs17683020	7	119,486	0,082	0,008							ETV1
rs7804006	7	151,751	0,205	0,015							inter
rs10486019	7	105,605	0,193	0,049							PBEF1
rs10950610	7	59,414	0,073	0,022							SOSTDC1
rs4141805	7	66,584	0,096	0,029							inter
rs2012007	7				43,669	0,043	0,014				inter
**rs6558435**	**8**	**236,327**	**0,468**	**0,041**	**181,398**	**0,150**	**0,011**				**ERICH1 (0.93) DLGAP2 (0.89)**
rs3134388	8							0,011	0,024	0,002	EXT1, THRAP6
rs1821134	8							147,730	0,188	0,023	PGCP, TSPYL5
rs1429948	8							105,479	0,079	0,006	ZMAT4
rs10107657	8							99,098	0,062	0,006	XKR4, TMEM68, NCOA6IP
rs2202989	8	0,005	0,299	0,028							SGCZ
rs7017160	8				0,008	0,132	0,017				FKSG2 (0.82)
rs710261	8				43,419	0,008	0,002				inter
rs2670882	8				100,903	0,004	0,000				DDEF1 (1.98)
rs2458064	8				109,850	0,138	0,021				CSMD3 (1.16)
rs10972238	9							0,005	0,052	0,000	inter
rs7045055	9							0,012	0,114	0,017	MPDZ
rs1332252	9							0,033	0,059	0,047	CDK5RAP2
rs17770971	9	124,472	0,104	0,011							LINGO2

rs874056	9	211,331	0,210	0,009							RAPGEF1
rs10967100	9	86,215	0,086	0,011							inter
rs4449884	9	0,010	0,105	0,020							inter
rs13439981	9	0,035	0,029	0,013							PGM5
rs10962181	9				0,018	0,053	0,013				inter
rs167565	9				92,006	0,118	0,025				C9orf84 (1.62)
rs10987199	9				147,109	0,144	0,010				FAM125B (1.92) PBX3 (5.44)
**rs10733833**	**10**	**136,688**	**0,205**	**0,033**				**176,217**	**0,269**	**0,027**	**CTNNA3, LRRTM3**
rs11008755	10							0,010	0,169	0,044	inter
rs4880401	10							0,014	0,049	0,008	C10orf91
rs12412248	10							0,016	0,052	0,009	MPP7
rs7477459	10							144,326	0,158	0,017	PHYHIPL
rs11814418	10							101,860	0,107	0,018	SHOC2
rs7904054	10							36,407	0,037	0,018	ADD3
rs17111156	10	60,928	0,102	0,042							ENTPD1
rs16917404	10	0,015	0,092	0,027							CACNB2
rs7075914	10	0,027	0,067	0,045							OLAH
rs12266178	10				31,430	0,006	0,000				CHAT (1.44)
rs11192335	10				36,732	0,037	0,011				SORCS3 (0.97)
rs2479037	10				47,379	0,019	0,002				VTI1A (0.94)
rs11191445	10				153,942	0,046	0,001				AS3MT (1.68)
rs11238595	10				167,382	0,117	0,003				ZNF239 (0.74) ZNF485 (0.57)
rs7087197	10				216,950	0,159	0,009				CNNM1 (1.41) GOT1 (1.02) HPSE2 (0.04)
**rs6589535**	**11**	**132,232**	**0,277**	**0,042**	**78,770**	**0,133**	**0,035**				**inter**
**rs10430848**	**11**	**254,632**	**0,439**	**0,028**	**148,169**	**0,187**	**0,026**				**TMEM16C (0.69)**
rs17129612	11							67,405	0,099	0,039	inter
rs16935076	11							60,038	0,087	0,026	MRGPRX3
rs1945063	11							58,751	0,026	0,001	GUCY1A2, AASDHPPT
rs16913217	11							43,479	0,028	0,004	NOX4
rs10840096	11	131,929	0,153	0,020							inter
rs10400310	11	108,532	0,135	0,017							inter
rs185932	11	43,213	0,069	0,042							inter
rs4287329	11	0,007	0,251	0,034							API5
rs7950582	11				38,557	0,055	0,023				ETS1 (1.75)
rs16930631	11				51,099	0,093	0,039				C11orf74 (1.55) RAG2 (1.13)
rs1998277	11				210,115	0,386	0,042				ABTB2 (0.40)
**rs2252178**	**12**	**194,081**	**0,413**	**0,035**	**168,457**	**0,333**	**0,044**				**inter**
rs10746146	12							0,005	0,240	0,012	PPP1R12A
rs11168355	12							0,013	0,129	0,029	PFKM, SENP1, COL2A1
rs7977296	12							0,014	0,086	0,012	TMEM132B
rs7135451	12							0,014	0,093	0,014	KLRD1, GABARAPL1
rs10507066	12							0,025	0,017	0,001	CCDC38
rs7308297	12	124,089	0,196	0,022							SYT1
rs3858650	12	145,658	0,178	0,020							inter
rs10778921	12	197,554	0,322	0,023							TMTC2
rs1565351	12	253,219	0,502	0,038							BTG1, DCN
rs12310279	12	90,151	0,117	0,022							inter
rs520242	12	45,897	0,029	0,007							PIK3C2G
rs994713	12	0,023	0,068	0,027							PKP2
rs11060323	12				0,021	0,084	0,037				TMEM132D (0.58)
rs17031072	12				38,120	0,050	0,013				SLC5A8 (0.77) TMEM16D (0.16)
rs10879757	12				47,581	0,074	0,034				inter
rs4761981	12				75,591	0,112	0,024				LOC57228 (1.28) BIN2 (1.27)
rs10743769	12				85,294	0,086	0,016				C12orf35 (0.80)
rs10506452	12				175,691	0,236	0,020				inter
rs9529872	13							0,102	0,020	0,045	DACH1
rs9580642	13							195,150	0,283	0,017	SACS, TNFRSF19
rs1929744	13	91,116	0,154	0,032							KCTD12
rs9552517	13	41,056	0,021	0,004							inter
rs1998833	13	0,041	0,043	0,041							ATP12A
rs17463402	13				0,005	0,306	0,017				inter
rs9548405	13				79,804	0,143	0,033				UFM1 (0.93) FREM2 (0.16)
rs7982903	13				97,510	0,197	0,045				KLF12 (1.36)
rs17083075	13				99,695	0,121	0,020				ATP8A2 (0.86) TMEM46 (2.95)

rs9591697	13				106,307	0,035	0,000				inter
SNP_A-4201974	13				113,749	0,038	0,002				inter
rs17056562	13				152,511	0,088	0,003				inter
rs7333155	13				191,214	0,051	0,001				RPL21 (1.58) USP12 (0.57)
**rs1548094**	**14**	**0,009**	**0,187**	**0,023**	**0,010**	**0,182**	**0,024**				**inter**
rs1950429	14							0,027	0,063	0,029	inter
rs1958682	14							0,029	0,055	0,022	STXBP6
rs944440	14							0,029	0,078	0,046	FAM12A, RNASE4, ANG
rs1548094	14	0,009	0,187	0,023							inter
rs17557187	14				0,020	0,076	0,020				inter
rs1383795	14				0,031	0,023	0,006				inter
rs7145954	14				41,942	0,070	0,023				TDP1 (0.42)
rs5024915	14				150,421	0,227	0,015				RNASE1 (3.50) RNASE6 (2.06)
rs712366	14				161,669	0,253	0,013				SLC25A21 (0.90)
rs16973230	15							0,006	0,093	0,000	MEX3B
rs17664311	15							0,035	0,025	0,009	inter
rs16963019	15							113,564	0,291	0,044	ATP8B4
rs2118181	15	154,118	0,419	0,038							FBN1
rs12161938	15	458,210	0,080	0,000							C15orf2
rs2959039	15	0,008	0,343	0,040							MTMR15
rs11071613	15				0,031	0,033	0,006				RORA (0.56) VPS13C (0.34)
rs8039109	15				0,046	0,032	0,011				inter
rs16957964	15				24,958	0,035	0,015				RYR3 (1.02)
rs11074316	15				224,747	0,512	0,030				GABRG3 (0.80)
**rs11865033**	**16**				**0,043**	**0,025**	**0,009**	**0,044**	**0,012**	**0,000**	**MAF (1.79)**
**rs11647011**	**16**	**384,064**	**0,183**	**0,003**	**323,569**	**0,196**	**0,000**				**CLEC16A (0.78)**
**rs322609**	**16**	**74,431**	**0,004**	**0,000**	**110,368**	**0,343**	**0,042**				**CDH11 (1.70)**
**rs16949415**	**16**	**0,015**	**0,099**	**0,019**	**0,020**	**0,098**	**0,021**				**WWOX (1.88)**
rs6500605	16							0,006	0,562	0,028	DNAJA3
rs2733954	16							0,007	0,184	0,012	COX4I1
rs7203742	16							0,011	0,068	0,005	CTCF
rs3910214	16							0,025	0,078	0,029	CDH13
rs16947741	16							75,844	0,150	0,021	ZNF423
rs16955369	16	41,020	0,088	0,032							AYTL1
rs16963958	16	43,201	0,081	0,035							CDH8
rs587388	16	0,008	0,391	0,041							FAM86A, ALG1
rs1362578	16	0,012	0,161	0,015							CBLN1
rs2982447	16				98,545	0,083	0,007				inter
rs7190066	16				181,207	0,516	0,036				TMC7 (1.16)
**rs2472694**	**17**	**82,014**	**0,159**	**0,030**	**81,709**	**0,223**	**0,035**				**GRAP (1.35) PRPSAP2 (0.73) SLC5A10 (1.36)**
rs11080041	17							0,010	0,103	0,004	CA10, KIF2B
rs17836935	17							130,317	0,222	0,012	RPL38, C17orf54
rs16970536	17							69,030	0,086	0,011	LIG3
rs16970084	17							63,565	0,120	0,015	TMEM132E
rs4362431	17				0,011	0,130	0,008				SP6 (1.65)
rs886078	17				69,014	0,199	0,048				CA10 (0.82)
rs2957385	17				71,199	0,192	0,028				CA10 (0.82) KIF2B (1.21)
rs17226067	17				76,980	0,133	0,014				inter
**rs10871642**	**18**				**54,143**	**0,111**	**0,038**	**50,092**	**0,108**	**0,039**	**CCDC102B (1.83)**
rs900968	18							0,010	0,222	0,027	SIGLEC15, SLC14A1
rs10782039	18							116,610	0,314	0,045	RTTN
rs16974452	18							102,010	0,231	0,042	APCDD1
rs572600	18	46,587	0,086	0,033							YES1
rs12455748	18	0,007	0,251	0,016							DCC
rs2156327	18				0,013	0,143	0,028				CYB5A (0.73)
rs16967025	18				0,029	0,053	0,021				GALNT1 (0.61)
rs16951910	18				47,892	0,074	0,021				PTPRM (1.06)
rs17797002	18				96,167	0,203	0,031				NETO1 (1.28)
rs12959798	18				291,798	0,582	0,023				CETN1 (1.01) COLEC12 (3.0)
**rs3746192**	**19**	**93,400**	**0,173**	**0,006**	**78,952**	**0,149**	**0,009**	**97,912**	**0,063**	**0,000**	**KCNN1 (1.02)**
rs6509349	19							143,835	0,535	0,032	SULT2A1
rs198963	19	77,112	0,057	0,000							KLK2
rs1105167	19	278,517	1,035	0,040							inter
rs17620912	19				0,011	0,219	0,016				inter
rs2965271	19				82,529	0,067	0,001				SAE2 (0.90)
**rs6049295**	**20**	**137,490**	**0,198**	**0,007**				**90,072**	**0,146**	**0,015**	**inter**
**rs1884779**	**20**	**141,503**	**0,362**	**0,031**	**118,669**	**0,360**	**0,035**				**SULF2 (1.42)**

rs1321338	20	144,823	0,430	0,044							MAFB
rs2756260	20				26,680	0,058	0,025				PRND (1.83) PRN (1.08)
rs6046214	20				89,287	0,140	0,021				SLC24A3 (0.34)
rs6056374	20				121,355	0,110	0,006				PLCB1 (0.10) PLCB4 (0.35)
rs6121430	20				162,388	0,386	0,029				CDH4 (0.81)
rs1041786	21							0,028	0,045	0,004	NCAM2
rs460014	21							280,793	0,357	0,003	inter
SNP_A-4215996	21							147,652	0,146	0,001	AGPAT3
rs11701130	21	0,006	0,178	0,003							SLC19A1, COL18A1, C21orf123
rs2824801	21	0,008	0,351	0,018							PRSS7
rs1709827	21	0,023	0,150	0,042							KCNJ6
**rs7291901**	**22**				**0,091**	**0,030**	**0,032**	**0,078**	**0,029**	**0,022**	**XPNPEP3 (0.49)**
rs2235165	22							0,021	0,133	0,030	FAM19A5
rs1811828	22							295,928	0,848	0,026	LRP5L
rs16980525	22	225,587	0,514	0,009							ADRBK2
rs6007763	22	16,076	0,048	0,022							RP11-191L9.1
**rs16986515**	**X**	**113,443**	**0,037**	**0,000**				**248,589**	**0,543**	**0,020**	**AMELX, ARHGAP6, MSL3L1**
rs16992781	X							271,567	0,801	0,035	inter
rs12008496	X							291,115	0,576	0,020	BCOR
rs5929497	X							150,700	0,242	0,013	inter
rs5913038	X	80,974	0,107	0,010							TBX22, ITM2A,
rs6632450	X	96,200	0,096	0,008							CXorf22
rs5975196	X	54,120	0,040	0,004							FAM45B, RBMX2
rs3761590	X	0,024	0,083	0,032							ITM2A
rs5936942	X	0,029	0,097	0,045							TEX11
rs5907594	X	0,042	0,064	0,037							SOX3
rs11266224	X				0,011	0,272	0,033				MID1IP1 (0.90)
rs16998540	X				46,396	0,092	0,019				inter

Supplemental table 5. Non-supervised gene functional clustering considering genes nearby the 280 top-ranked SNPs.

**DAVID CLUSTERING FOR TOP-RANKED SNPs**	
**"transmembrane, adhesion, cadherin" (Enrichment Score: 2.13)**	
NCAM2	neural cell adhesion molecule 2
CDH12	cadherin 12, type 2 (n-cadherin 2)
CDH4	cadherin 4, type 1, r-cadherin (retinal)
PCDH7	bh-protocadherin (brain-heart)
CDH8	cadherin 8, type 2
CDH11	cadherin 11, type 2, ob-cadherin (osteoblast)
FREM2	fras1 related extracellular matrix protein 2
RYK	ryk receptor-like tyrosine kinase
CNNM1	cyclin m1
PAQR3	progestin and adipoq receptor family member iii
ROBO2	roundabout, axon guidance receptor, homolog 2 (drosophila)
ANTXR2	anthrax toxin receptor 2
GPR63	g protein-coupled receptor 63
NETO1	neuropilin (nrp) and tolloid (tll)-like 1
NCR2	natural cytotoxicity triggering receptor 2
TNFRSF19	tumor necrosis factor receptor superfamily, member 19
GPR156	g protein-coupled receptor 156
MRGPRX3	mas-related gpr, member x3
BTLA	b and t lymphocyte associated
PTPRM	protein tyrosine phosphatase, receptor type, m
KLRD1	killer cell lectin-like receptor subfamily d, member 1
IL1RL2	interleukin 1 receptor-like 2
IL23R	interleukin 23 receptor
DCC	deleted in colorectal carcinoma
SORCS3	sortilin-related vps10 domain containing receptor 3
TRAM2	translocation associated membrane protein 2
EMCN	endomucin
SDK1	sidekick homolog 1 (chicken)
SLC14A1	solute carrier family 14 (urea transporter), member 1 (kidd blood group)
SLC19A1	solute carrier family 19 (folate transporter), member 1
CD200	cd200 antigen
ITM2A	integral membrane protein 2a
LRRTM3	leucine rich repeat transmembrane neuronal 3
TMEM33	transmembrane protein 33
SGCZ	sarcoglycan zeta
MGC33214	hypothetical protein mgc33214
XKR4	xk, kell blood group complex subunit-related family, member 4
TMEM16C	transmembrane protein 16c
APCDD1	adenomatosis polyposis coli down-regulated 1
TMEM132E	transmembrane protein 132e

**"ribonuclease" (Enrichment Score: 2.02)**	
RNASE1	ribonuclease, rnase a family, 1 (pancreatic)
RNASE4	ribonuclease, rnase a family, 4
RNASE6	ribonuclease, rnase a family, k6
FAM12A	family with sequence similarity 12, member a
**"ion channel transport" (Enrichment Score: 1.64)**	
KCND2	potassium voltage-gated channel, shal-related subfamily, member 2
RYR2	ryanodine receptor 2 (cardiac)
RYR3	ryanodine receptor 3
CACNB2	calcium channel, voltage-dependent, beta 2 subunit
KCNJ6	potassium inwardly-rectifying channel, subfamily j, member 6
KCTD12	potassium channel tetramerisation domain containing 12
KCNN1	potassium intermediate/small conductance calcium-activated channel, subfamily n, member 1
**"Golgi apparatus" (Enrichment Score: 1.12)**	
EXT1	exostoses (multiple) 1
MAN1A1	mannosidase, alpha, class 1a, member 1
NDST4	n-deacetylase/n-sulfotransferase (heparan glucosaminyl) 4
ST6GALNAC5	st6 (alpha-n-acetyl-neuraminyl-2,3-beta-galactosyl-1,3)-n-acetylgalactosaminide alpha-2,6-sialyltransferase 5
NDST3	n-deacetylase/n-sulfotransferase (heparan glucosaminyl) 3
GALNT1	udp-n-acetyl-alpha-d-galactosamine:polypeptide n-acetylgalactosaminyltransferase 1 (galnac-t1)
MGAT1	mannosyl (alpha-1,3-)-glycoprotein beta-1,2-n-acetylglucosaminyltransferase
ALG1	asparagine-linked glycosylation 1 homolog (yeast, beta-1,4-mannosyltransferase)

**DAVID CLUSTRING FOR SNPs ASSOCIATED WITH DIE**	
**"transmembrane" (Enrichment Score: 1.89)**	
SDK1	sidekick homolog 1 (chicken)
TMEM33	transmembrane protein 33
MGC33214	hypothetical protein mgc33214
XKR4	xk, kell blood group complex subunit-related family, member 4
APCDD1	adenomatosis polyposis coli down-regulated 1
PAQR3	progestin and adipoq receptor family member iii
LRRTM3	leucine rich repeat transmembrane neuronal 3
NCR2	natural cytotoxicity triggering receptor 2
IL23R	interleukin 23 receptor
TMEM132E	transmembrane protein 132e
**"transmembrane" (Enrichment Score: 1.23)**	
GPR156	g protein-coupled receptor 156
MRGPRX3	mas-related gpr, member x3
EMCN	endomucin
GPR63	g protein-coupled receptor 63
KLRD1	killer cell lectin-like receptor subfamily d, member 1
PAQR3	progestin and adipoq receptor family member iii
NCR2	natural cytotoxicity triggering receptor 2
TNFRSF19	tumor necrosis factor receptor superfamily, member 19

DAVID CLUSTERING FOR SNPs ASSOCIATED WITH OMA	
**"transmembrane, cell adhesion, cadherin" (Enrichment Score: 0.89)**	
NETO1	neuropilin (nrp) and tolloid (tll)-like 1
FREM2	fras1 related extracellular matrix protein 2
BTLA	b and t lymphocyte associated
PTPRM	protein tyrosine phosphatase, receptor type, m
CDH4	cadherin 4, type 1, r-cadherin (retinal)
CDH11	cadherin 11, type 2, ob-cadherin (osteoblast)
SDK1	sidekick homolog 1 (chicken)
CDH12	cadherin 12, type 2 (n-cadherin 2)
SORCS3	sortilin-related vps10 domain containing receptor 3
GPR63	g protein-coupled receptor 63
PAQR3	progestin and adipoq receptor family member iii
CD200	cd200 antigen
**"regulation of transcription, DNA binding, zinc finger" (Enrichment Score: 0.83)**	
RUNX2	runt-related transcription factor 2
HSF2	heat shock transcription factor 2
KLF12	kruppel-like factor 12
DLGAP2	discs, large (drosophila) homolog-associated protein 2
CDC5L	cdc5 cell division cycle 5-like (s. pombe)
ZNF485	zinc finger protein 485
ETS1	v-ets erythroblastosis virus e26 oncogene homolog 1 (avian)
MAF	v-maf musculoaponeurotic fibrosarcoma oncogene homolog (avian)
PBX3	pre-b-cell leukemia transcription factor 3
ZNF680	zinc finger protein 680
SUPT3H	suppressor of ty 3 homolog (s. cerevisiae)
SP6	sp6 transcription factor
ZNF366	zinc finger protein 366
HIVEP1	human immunodeficiency virus type i enhancer binding protein 1
RORA	rar-related orphan receptor a
ZNF239	zinc finger protein 239
POLR2B	polymerase (rna) ii (dna directed) polypeptide b, 140kda
ZNF659	zinc finger protein 659

DAVID CLUSTERING FOR SNPs ASSOCIATED WITH SUP	
**"cadherin, cell adhesion" (Enrichment Score: 1.44)**	
ANTXR2	anthrax toxin receptor 2
PCDH7	bh-protocadherin (brain-heart)
CDH8	cadherin 8, type 2
CDH11	cadherin 11, type 2, ob-cadherin (osteoblast)
SGCZ	sarcoglycan zeta
**"ion channel transport" (Enrichment Score: 1.08)**	
KCNN1	potassium intermediate/small conductance calcium-activated channel, subfamily n, member 1
KCTD12	potassium channel tetramerisation domain containing 12
CACNB2	calcium channel, voltage-dependent, beta 2 subunit

KCNJ6	potassium inwardly-rectifying channel, subfamily j, member 6
**"golgi apparatus" (Enrichment Score: 0.98)**	
ST6GALNAC5	st6 (alpha-n-acetyl-neuraminyl-2,3-beta-galactosyl-1,3)-n-acetylgalactosaminide alpha-2,6-sialyltransferase 5
ALG1	asparagine-linked glycosylation 1 homolog (yeast, beta-1,4-mannosyltransferase)
NDST4	n-deacetylase/n-sulfotransferase (heparan glucosaminyl) 4
NDST3	n-deacetylase/n-sulfotransferase (heparan glucosaminyl) 3
MGAT1	mannosyl (alpha-1,3-)-glycoprotein beta-1,2-n-acetylglucosaminyltransferase

**DAVID CLUSTERING FOR SNPs ASSOCIATED WITH SUP, OMA and DIE**	
**"Transmembrane" (Enrichment Score: 0.73)**	
TMEM16C	transmembrane protein 16c
TMEM33	transmembrane protein 33
ITM2A	integral membrane protein 2a
PAQR3	progestin and adipoq receptor family member iii
LRRTM3	leucine rich repeat transmembrane neuronal 3

Supplemental table 6. Distribution of genotype frequencies in cases and controls. NA: not applicable, OR: odds ratio, 95%CI: 95% confidence interval, CTR: controls, SUP: peritoneal superficial endometriosis, OMA: endometrioma, DIE: deep infiltrating endometriosis. P-values are for the Pearson's chi-square test.

	CTR			SUP			p-value	OMA			p-value	DIE			p-value
rs776108	AA	AG	GG	AA	AG	GG		AA	AG	GG		AA	AG	GG	
	52	34	4	15	6	1	0.648	30	13	1	0.480	50	32	2	0.756
rs3746192	AA	AG	GG	AA	AG	GG		AA	AG	GG		AA	AG	GG	
	4	28	59	0	15	26	0.349	3	17	30	0.822	7	35	54	0.431
rs988147	CC	CT	TT	CC	CT	TT		CC	CT	TT		CC	CT	TT	
	29	47	19	13	18	8	0.934	33	16	5	0.061	29	42	14	0.785
rs227849	AA	AG	GG	AA	AG	GG		AA	AG	GG		AA	AG	GG	
	39	39	17	9	22	11	0.081	7	27	16	0.003	40	39	17	0.996
rs10733833	CC	CG	GG	CC	CG	GG		CC	CG	GG		CC	CG	GG	
	13	41	38	8	21	13	0.486	6	26	18	0.697	17	43	35	0.720
rs322609	CC	CT	TT	CC	CT	TT		CC	CT	TT		CC	CT	TT	
	42	38	14	14	22	6	0.395	15	27	7	0.209	33	47	16	0.342
rs1884779	AA	AG	GG	AA	AG	GG		AA	AG	GG		AA	AG	GG	
	28	47	19	18	16	5	0.179	17	23	8	0.757	34	42	20	0.648
rs4703908	CC	CG	GG	CC	CG	GG		CC	CG	GG		CC	CG	GG	
	64	30	1	22	18	2	0.140	24	21	5	0.009	56	39	1	0.427
rs6946871	CC	CT	TT	CC	CT	TT		CC	CT	TT		CC	CT	TT	
	3	26	65	2	9	31	0.697	3	18	29	0.371	4	30	62	0.787
rs11865033	CC	CG	GG	CC	CG	GG		CC	CG	GG		CC	CG	GG	
	0	18	75	0	6	36	0.476	0	12	38	0.515	0	11	83	0.148
rs247004	AA	AG	GG	AA	AG	GG		AA	AG	GG		AA	AG	GG	
	1	20	50	0	8	28	0.604	0	12	34	0.691	1	23	58	0.994
rs11647011	AA	AG	GG	AA	AG	GG		AA	AG	GG		AA	AG	GG	
	66	13	2	34	7	0	0.595	36	8	1	0.967	74	11	1	0.667
rs7477459	CC	CG	GG	CC	CG	GG		CC	CG	GG		CC	CG	GG	
	64	21	1	23	9	1	0.724	37	8	0	0.510	58	11	1	0.407
rs5913038	AA	AG	GG	AA	AG	GG		AA	AG	GG		AA	AG	GG	
	67	27	1	29	10	0	0.762	32	9	1	0.600	64	30	2	0.758
rs17056959	CC	CT	TT	CC	CT	TT		CC	CT	TT		CC	CT	TT	
	0	6	77	0	4	37	0.627	0	5	40	0.454	0	4	69	0.656
rs16986515	CC	CT	TT	CC	CT	TT		CC	CT	TT		CC	CT	TT	
	94	0	0	39	0	0	NA	44	0	0	NA	96	0	0	NA

Supplemental table 7. Distribution of genotype frequencies in cases and controls for the DIE subtypes. NA: not applicable, OR: odds ratio, 95%CI: 95% confidence interval, CTR: controls, USL: uterosacral ligaments, VA: vagina, BLA: bladder, DIG: intestine. P-values are for the Pearson's chi-square test.

	CTR			USL			p-value	VA			p-value	BLA			p-value	DIG			p-value
rs776108	AA	AG	GG	AA	AG	GG		AA	AG	GG		AA	AG	GG		AA	AG	GG	
	52	34	4	9	10	2	0.383	15	5	0	0.296	8	4	0	0.694	18	13	0	0.477
rs3746192	AA	AG	GG	AA	AG	GG		AA	AG	GG		AA	AG	GG		AA	AG	GG	
	4	28	59	1	9	14	0.820	2	5	16	0.542	2	5	5	0.132	2	16	19	0.312
rs988147	CC	CT	TT	CC	CT	TT		CC	CT	TT		CC	CT	TT		CC	CT	TT	
	29	47	19	8	12	2	0.480	8	9	3	0.689	0	4	3	0.150	13	17	6	0.805
rs227849	AA	AG	GG	AA	AG	GG		AA	AG	GG		AA	AG	GG		AA	AG	GG	
	39	39	17	9	10	5	0.927	13	6	4	0.351	4	6	2	0.831	14	17	6	0.877
rs10733833	CC	CG	GG	CC	CG	GG		CC	CG	GG		CC	CG	GG		CC	CG	GG	
	13	41	38	3	12	8	0.802	4	11	8	0.831	3	4	5	0.570	7	16	14	0.787
rs322609	CC	CT	TT	CC	CT	TT		CC	CT	TT		CC	CT	TT		CC	CT	TT	
	42	38	14	5	16	3	0.058	9	13	1	0.243	4	4	4	0.275	15	14	8	0.649
rs1884779	AA	AG	GG	AA	AG	GG		AA	AG	GG		AA	AG	GG		AA	AG	GG	
	28	47	19	6	11	7	0.633	9	9	5	0.610	4	6	2	0.946	15	16	6	0.494
rs4703908	CC	CG	GG	CC	CG	GG		CC	CG	GG		CC	CG	GG		CC	CG	GG	
	64	30	1	18	6	9	0.709	12	10	1	0.269	11	1	0	0.222	15	22	0	0.012
rs6946871	CC	CT	TT	CC	CT	TT		CC	CT	TT		CC	CT	TT		CC	CT	TT	
	3	26	65	2	6	16	0.533	1	10	12	0.304	0	5	7	0.533	1	9	27	0.911
rs11865033	CC	CG	GG	CC	CG	GG		CC	CG	GG		CC	CG	GG		CC	CG	GG	
	0	18	75	0	1	23	0.072	0	2	21	0.226	0	1	10	0.405	0	7	29	0.991
rs247004	AA	AG	GG	AA	AG	GG		AA	AG	GG		AA	AG	GG		AA	AG	GG	
	1	20	50	0	5	17	0.739	0	10	11	0.226	0	2	10	0.632	1	6	20	0.670
rs11647011	AA	AG	GG	AA	AG	GG		AA	AG	GG		AA	AG	GG		AA	AG	GG	
	66	13	2	22	0	0	0.092	16	4	1	0.800	8	1	0	0.817	28	6	0	0.644
rs7477459	CC	CG	GG	CC	CG	GG		CC	CG	GG		CC	CG	GG		CC	CG	GG	
	64	21	1	15	3	0	0.686	13	4	1	0.465	5	0	0	0.430	25	4	0	0.396
rs5913038	AA	AG	GG	AA	AG	GG		AA	AG	GG		AA	AG	GG		AA	AG	GG	
	67	27	1	18	5	1	0.456	15	8	0	0.752	7	4	1	0.188	24	13	0	0.635
rs17056959	CC	CT	TT	CC	CT	TT		CC	CT	TT		CC	CT	TT		CC	CT	TT	
	0	6	77	0	1	15	0.889	0	1	16	0.843	0	2	6	0.090	0	0	32	0.118
rs16986515	CC	CT	TT	CC	CT	TT		CC	CT	TT		CC	CT	TT		CC	CT	TT	
	94	0	0	24	0	0	NA	23	0	0	NA	12	0	0	NA	37	0	0	NA

Supplemental table 8. Distribution of genotype and allele frequencies in cases and controls. Testing deviation from the Hardy Weinberg proportions (HWP) was performed using Pearson's chi-squared test (with a 5% significance level for 1 degree of freedom at 3.84). OR: odds ratio, 95%CI: 95% confidence interval. P-values are for the Pearson's chi-squared test.

	Genotypes			Alleles		χ2 test for HWP deviation	OR	95%CI	P-value
rs16913217	CC	CT	TT	C (%)	T (%)				
OMA	0	1	70	0.7	99.3	0,998			
Controls	1	11	180	3.4	96.4	0,227	4.94	0.64-38.12	0.270
rs2479037	CC	CT	TT	C (%)	T (%)				
OMA	0	3	63	2.3	97.7	0,982			
Controls	5	25	160	9.2	90.8	0,013	4.36	1.32-14.43	0.005
rs966674	CC	CG	GG	C (%)	G (%)				
OMA	50	17	3	83.6	16.4	0,627			
Controls	169	22	1	93.7	6.3	0,954	2.95	1.60-5.42	0.002
rs7333155	AA	AG	GG	A (%)	G (%)				
OMA	63	7	0	95.0	5.0	0,908			
Controls	156	17	8	90.9	9.1	0,000	0.52	0.23-1.22	0.202
rs11865033	CC	CG	GG	C (%)	G (%)				
OMA	0	15	54	10.9	89.1	0,599			
Controls	0	28	142	8.2	91.8	0,504	0.74	0.38-1.43	0.337
